# Progesterone receptor A and c-Met mediates spheroids-endometrium attachment

**DOI:** 10.1186/1477-7827-7-14

**Published:** 2009-02-16

**Authors:** Haggar Harduf, Shlomit Goldman, Eliezer Shalev

**Affiliations:** 1 Laboratory for Research in Reproductive Sciences, Department of Obstetrics and Gynecology, Ha'Emek Medical Center, Afula, Israel; 2 Rappaport Faculty of Medicine, Technion-Israel Institute of Technology, Haifa, Israel

## Abstract

**Background:**

Implantation in humans involves cross talk between an active blastocyst and receptive endometrium. The role of the endometrial receptors in this complex embryo-maternal interaction is still unclear. We tested gene and protein expression of endometrial receptors (Progesterone receptor (PR) and c-Met) and the effect of theses receptors in endometrial receptivity.

**Methods:**

Two endometrial cell lines were used: HEC-1A and RL95-2 considered as being of low and high receptivity, respectively. Western blot and RT-PCR analysis were utilized to study the receptor expression profile.

The role of endometrial receptors in endometrial receptivity was studied by attachment and invasion assays of JAR spheroids (made of a trophoblast cell line) on endometrial cells. Different manipulations of inhibition and stimulation of the endometrial receptors were used including: inhibition by specific antibodies against the receptors, or antagonist of the receptors, as well as transfection with antisense for the endometrial receptors, stimulation by specific ligands for the receptors and transfection with the gene for endometrial receptors.

**Results:**

Different protein expression patterns of endometrial receptors were observed between the tested endometrial cell lines. The expression levels of PRA ratio to PRB, and the 50 kDa c-MET isoform were significantly lower in HEC-1A as compared with RL95-2. Attachment rates and growth of JAR spheroids into HEC-1A were significantly lower as compared with RL95-2. Stimulation of PR with progesterone altered attachment rates to HEC-1A. Inhibition of PR with RU-486 mildly increased attachment rate to HEC-1A whereas it slightly decreased attachment rate to RL95-2. c-Met inhibition decreased attachment rates only to HEC-1A cells that expressing high levels of Plexin-B1 (PB1). Immunoprecipitation studies revealed that c-Met and PB1 associate in complexes in the endometrial cell lines.

**Conclusion:**

Differential endometrial receptor profiles are expressed during the receptivity period. The attachment and invasion processes are separately regulated. We suggest a biologically functional role for PRA in endometrial receptivity and in the attachment process. c-Met contribution is minor and related with creation of a complex with PB1.

## Background

Implantation in humans involves complex interactions between the embryo and the maternal endometrium [1-3]. Successful implantation depends on a pre-implantation embryo developing into a competent blastocyst that reaching the uterus precisely at its receptive stage [4]. Endometrial receptivity is suggested to be a property of the endometrial epithelial cells (EECs). The molecular mechanisms by which the surface of human EECs acquires morphological changes, leading to receptive features, are still unclear. Cytokines, growth factors, hormones, extracellular matrix proteins and enzymes, angiogenic factors, cell-cell adhesion molecules and receptors are all involved in this complex process [5]. Previous studies demonstrated the appearance of morphological or biological markers for endometrial receptivity [6-10]. However functional physiological markers are still unknown. The cross talk, between the active blastocyst and the receptive uterus, is solely reliant on mediation and interrelationship by a variety of receptors in the endometrium. Despite the possibility of extra corporal fertilization and extensive new technology, the process of implantation and the interaction between maternal endometrium and invading trophoblast are even today difficult to explore.

Hence, the search for better understanding of this process continues and is transferred into the in vitro setting [11-13]. In our previous study [14] we showed that Plexin B1 (PB1), a membrane receptor, has a role in endometrial receptivity and in the attachment process. The current study was designed to explore and compare the expression and role of the membrane receptor c-Met, which is known to be expressed as a complex with PB1 [15,16] and the nuclear receptor PR in two human endometrial cell lines, RL95-2 and HEC-1A, used as a model for high receptivity and low receptivity endometrium respectively [17-20].

The progesterone receptor (PR) is a member of a large family of ligand-activated nuclear transcription regulators, which are characterized by organization into specific functional domains and are conserved between species and family members. The PR is made up of a central DNA binding domain and a carboxyl-terminal ligand-binding domain. Studies on human PR indicate that there are at list 3 different alternatively spliced forms to the PR. Two of the PR isoforms, namely PR-A and PR-B, mediate the effects of progesterone. Detailed function studies indicate that PR-B, in all cellular contexts in-vitro, functions as a ligand-dependent trans-activator. This in contrast to PR-A, which in some contexts acts as a ligand-dependent transcriptional repressor of PR-B [21,22]. There is increasing evidence to date that PR-A and PR-B are functionally different. The PRB/PRA ratio was found to be of clinical importance in several tissues, [[23], and [24]]. These differences are yet to be fully understood. It is the balance between these two forms that may make it possible for progesterone to affect such diverse physiological targets. Progesterone's action has been shown to be essential for proper endometrial maturation, endometrial receptivity and the maintenance of pregnancy [25]. These effects of progesterone are thought to be mediated primarily through its cognate receptor [21,22]. The establishment of normal endometrial receptivity appears to be closely associated with the down-regulation of epithelial PR [8]. Histologic delay is associated with a failure of PR down-regulation and the lack of normal markers of endometrial receptivity.

The proto-oncogene Met encodes a transmembrane tyrosin kinase of 190 kDa. c-Met is a heterodimer composed of two disulfide-linked chains of 50 kDa and 140 kDa [26]. Met is the receptor for hepatocyte growth factor (HGF) [26-28]. It is frequently over expressed in neoplastic cells and in host tissue. Due to its prominent role in the control of motility and invasion, it is involved in metastasis formation. The role of c-Met in endometrial receptivity still needs to be investigated.

Stromal and trophoblast cells produce HGF [29] while its receptor is expressed in the endometrial epithelia and stroma [30]. Recent data indicate that signaling activity of the Met receptor is affected by an association with other receptors such as RON and PB1 and it was published that cells expressing the endogenous proteins, PB1 and c-Met, associate in a complex [15]. In addition it was shown that membrane-bound semaphorin Sema4D, PB1's ligand, can trigger the activation of the oncogenic receptor Met, which is associated with PB1 on the cell surface [16].

## Methods

### Cell lines

Two endometrial cell lines were used as in vitro model for endometrial receptivity. Cell line RL95-2 (ATCC catalog No.CRL-1671), derived from a moderately differentiated adeno-squamous carcinoma of the endometrium [17] was used as a model for receptive endometrium [19,20] Cell line HEC-1A (ATCC catalog No. HTB-112) derived from human endometrial carcinoma, served as a model for the non-receptive state [31]. Third cell line was established in our laboratory, HEC-1A cells were transfected with human PB1 (was provided kindly by Prof. Luca Tamagnone, the transfected cell line named HEC-1A-2. (HEC-1A-2 was characterized in our previous study [14]. Human trophoblast cell line, JAR (ATCC catalog No. HTB-144) was used as a model for blastocysts.

### Endometrial cell culture

HEC-1A cells were cultured in Meckoy 5A medium (Kibbutz Beit-Ha'Emek, Israel) containing 10% Fetal Calf Serum (FCS) (Kibbutz Beit-Ha'Emek, Israel) and penicillin/streptomycin (Kibbutz Beit-Ha'Emek, Israel) [32]. RL95-2 cells were cultured in DMEM F: 12 medium (Kibbutz Beit-Ha'Emek, Israel) containing FCS, penicillin/streptomycin, 2.5 mM Glutamine (Kibbutz Beit-Ha'Emek, Israel) [17]. Cell cultures were maintained in a humidified atmosphere containing 5% CO_2 _at 37°C. RL95-2 cells (1–2 × 10^6^) and HEC-1-A cells (1–2 × 10^6^) were seeded in 24-well culture plates for 10 days, and the growth medium was renewed every 2–3 days. All studies performed with serum free medium.

### Attachment and growth assays

#### Attachment of JAR spheroids to endometrial cell monolayer

For the attachment assays JAR spheroids were prepared and tested as described in details elsewhere [33]: briefly, 1 × 10^6 ^JAR cells per 10 ml M-199 medium (Kibbutz Beit-Ha'Emek, Israel) containing 10% FCS and penicillin/streptomycin were agitated at 37°C on a Comfort shaker at 200 rpm (12 × g). In order to distinguish JAR spheroids from underlying endometrial cell lines (HEC-1A and RL95-2) or primary culture we have labeled the JAR spheroids with the membrane-permeable fluorescent dye CMFDA (Invitrogen, Dorset, UK) that after enzymatic cleavage serves as a long-term cytoplasmic marker. Spheroids were agitated at 37°C for 24 hours. Thereafter spheroids were gently delivered with micro denuding pipette (150 μm diameter) onto a confluent monolayer of endometrial cell lines (HEC-1A and RL95-2) grown in 24-wells culture plates in M-199 growth medium containing 1.5% FCS. After 60 minutes of incubation at 37°C the culture plate was shaken aggressively at 15 × g (VORTEX GENIE, Scientific Industries, Chicago, U.S.A) for 60 minutes. The medium containing unattached spheroids was collected, and fresh medium was added to the wells. Spheroids remaining in each well were counted using a phase-contrast microscope or florescence microscope. Spheroids attachment is expressed as a percentage of seeded spheroids. In certain experiments HEC-1A and RL95-2 cell lines were pretreated with Progesterone 0–10 μM (Sigma, ST Louis, MO, USA) or with RU-486 (PR antagonist) (Sigma, ST Louis, MO, USA). In other experiments endometrial cell lines were pretreated with antisense against c-Met (IDT Inc, Hy-Labs, Rehovot, Israel).

#### Growth of JAR spheroids in endometrial cell monolayer

Spheroids outgrowth was measured under the microscope for the next 10 days. Each spheroid diameter size was measured using a special scale in the ocular.

### Preparation of whole cell extract and western blot analysis

HEC-1A and RL95-2 cells were lysed on ice in lysis buffer (20 mM Tris-HCl, pH 7.4, 5 mM EDTA, 150 mM NaCl, 10% glycerol, 1% Triton X-100) in the presence of a mixture of protease inhibitors (Roche, Kulmbach, Germany), suspensions were incubated for 7 minutes in 4°C. Cell lysates were precleared by centrifugation at 12000 rpm for 20 minutes, the supernatant fraction contained proteins.

### Protein assay

The total protein content of endometrial cells was determined using a protein assay kit with BSA as the standard (Bio-Rad laboratories, Inc, Washington DC). One to five microliters of sample were used in the assay. The assay is based on the Bradford dye-binding procedure.

### Western blot

In order to detect c-Met and PR, whole cell and nuclear extracts were diluted with 4 × sample buffer (5% SDS, 20% Glycerol in 0.4 M Tris, pH 6.8 containing 0.02% bromophenol blue) and subjected to 8% polyacrylamide gel electrophoresis. After electrophoresis, the proteins (50 μg/lane) were blotted from the SDS-PAGE onto 0.45 μm nitrocellulose membranes (Schleicher & Schuell, Dassel, Germany). Nonspecific binding sites were blocked by incubating the nitrocellulose membranes for 1 hour with 5% BSA (Sigma, ST Louis, MO, USA) in Tris-buffered saline. The membranes were then washed four times with Tris-buffered saline, containing 0.75% Tween-20, and incubated for 1 hour with antibodies against PR (Santa Cruz, Biotech, California, USA sc-539) or c-Met (Santa Cruz Biotech, California, USA sc-161) in 0.5% BSA in Tris-buffered saline, containing 0.01% Tween-20. The membranes were subsequently washed with Tris-buffered saline, containing 0.75% Tween-20 and incubated for 1 hour with HRP-conjugated Horseradish peroxidase-conjugated goat anti- rabbit secondary antibody (Jackson Immuno-research, Enco, Israel) in 0.5% BSA in Tris-buffered saline, containing 0.01% Tween-20. Proteins were detected by enhanced chemiluminescence (ECL Kit, Kibbutz Beit-Ha'Emek, Israel,) and quantified using the BioImaging gel documentation system (Dinco & Renium, Jerusalem, Israel) endowed with TINA software (Raytest, Taubenhardt, Germany). PR and c-Met were expressed as percent of control. For normalization we have used the levels of the housekeeping protein GAPDH.

### Semiquantitative RT-PCR

To analyze the expression of PR and c-Met, total RNA was prepared from cell cultures with EZ-RNA Kit (Kibbutz Beit-Ha'Emek, Israel). RNA concentrations were determined spectrophotometrically. To obtain the cDNA from cell lines, total RNA (5 μg) was denatured at 70°C for 10 min and then reverse transcribed in the presence of 25 ng/μl random primer (Promega, Mannheim, Germany), 2.5 mM MgCl_2_, 0.5 mM deoxy-NTPs, 10 mM dithiothreitol, and 10 U ribonuclease H- reverse transcriptase (Superscript II RT, Life Technologies, Inc.) for 60 min at 42°C, and 5 min at 95°C. Subsequently, 10 μl of the resulting cDNA was used as a template for polymerase chain reaction (PCR). The PCR was set up using 3 mM MgCl_2_, 50 pmol of each primer and 2.5 U Taq DNA polymerase (Sigma, St. Louis, MO, USA).

Primer design: the sequences of the primers were taken from the Genbank

PRB (Genbank access no. M15716)

PRB FWD 5'- ACACCTTGCCTGAAGTTTCG-3'

PRB REV 5'- CTGTCCTTTTCTGGGGGACT-3' (196 bp product).

c-MET – (Genbank access no. M35073)

c-MET FWD 5'- CTACAAAGAAGTTGATGAACCG-3'

c-MET REV 5'- GCTGACATACAGTCGGAGG-3' (139 bp product).

For normalization we have used the levels of the housekeeping gene *GAPDH*.

GAPDH FWD 5'-TGATGACATCAAGAAGGTGGTGAAG-3'; GAPDH REV 5'-TCCTTGGAGGCCATGTGGGCCAT-3' (230 bp product).

PCR conditions were 94°C for 2 min followed by 35 cycles of 94°C for 30 sec, 58°C for 45 sec, and 72°C for 60 sec with a 72°C extension for 10 min. After PCR, the products were resolved on a 2.5% agarose ethidium bromide gel. Images were captured with Polaroid (Hertfordshire, UK) film under UV light. Products were quantified using PhosphorImager and ImageQuant software (Molecular Dynamics, Inc., Sunnyvale, CA).

### Immunoprecipitation

Endometrial cell lines (HEC-1A, RL95-2, HEC-1A-2) were washed twice in ice cold PBS and lysed on ice in lysis buffer (20 mM Tris-HCl, pH 7.4, 5 mM EDTA, 150 mM NaCl, 10% glycerol, 1% Triton X-100) in the presence of a mixture of protease inhibitors (Roche, Kulmbach, Germany). 500 μg of whole cell extract in 1 ml lysis buffer were subject for immunoprecipitation and PB1 receptors were immunoprecipitated by incubation for 2 h on rocker at 4°C with 1 μg anti-PB1 antibody (Santa Cruz Biotech, California, USA, sc-28372). Immunocomplexes were recovered with the aid of 20 μl protein A/G agarose beads (Santa Cruz Biotech, California, USA, sc-2003). Each sample was placed on a rocker at 4°C for 1 h and thereafter incubated for 16 h at 4°C. The beads were washed twice with 1 ml lysis buffer and twice with Tris-EDTA (TE) and subsequently the bound proteins were eluted in 50 μl of 1% SDS in TE. Sample buffer was added to the supernatant of each sample. Lysates and immunoprecipitates were analyzed by Western blotting after SDS-polyacrylamide gel electrophoresis and transfer to a 0.45 μm nitrocellulose membranes (Schleicher & Schuell, Dassel, Germany) with anti c-Met antibodies (Santa Cruz Biotech, California, USA, sc-161). Proteins were detected by enhanced chemiluminescence (ECL Kit, Kibbutz Beit-Ha'Emek, Israel). As a negative control, PB1 immunoprecipitation was performed, followed by Western blotting with GAPDH antibody.

### Immunofluorescence staining

For immunofluorescence analysis, endometrial cells were cultured on glass coverslips in 35 μl medium drops under mineral oil. Cells were washed 3 times with PBS and fixed with 3.7% paraformaldehyde (Electron Microscope Sciences, Belgar) in PBS for 10 minutes at 4°C, then washed twice with PBS and permeabilized for 5 minutes at 4°C with 0.1% Triton (Sigma, St. Louis, MO, USA) in PBS. After a PBS wash, slides were incubated for 1 hour with blocking buffer (PBS supplemented with 3% BSA), then washed 3 times with PBS and incubated for 30 minutes at room temperature with primary antibodies (Anti c-Met), 1 μg per slide in 700 μl PBS supplemented with 1.5% BSA. After five washings with PBS, slides were incubated for 30 minutes in the dark with secondary fluorescein-labeled antibody (for F-actin:phalloidin, AlexaFlour-488, A-12379; for c-Met: goat anti-rabbit IgG conjugated with AlexaFlour-546, all from Molecular Probes, Invitrogen, Dorset, UK), 0.5 μg per slide in 700 μl PBS supplemented with 1.5% BSA. Following three washings with PBS, stained cells were photographed using a confocal microscope (the confocal system is composed of a Bio-Rad radiance 2000 confocal set-up hooked to an upright fluorescent microscope Nikon E600 with a 60X lens). The photos were analyzed by Image Pro software (Media Cybernetics, Bethesda, USA), which quantifies density per area.

### Statistical analysis

Results are expressed as mean ± SEM, with n denoting the number of spheroids. Student's t-test, chi test and "one way analysis of variance" (ANOVA) were used when appropriate. P < 0.05 was considered significant.

## Results

### PR expression in RL95-2 and HEC-1A cells

PRB gene expression was studied by RT-PCR. For normalization we have used the levels of the housekeeping gene GAPDH. In order to exclude the possibility of fluctuation in gene expression during 24 hours (h) period, we have studied the basal PRB gene expression on 2, 12 and 24 h of incubation with serum-free medium, 2 h after medium replacement considered as starting period (time 0).

**Figure 1:**
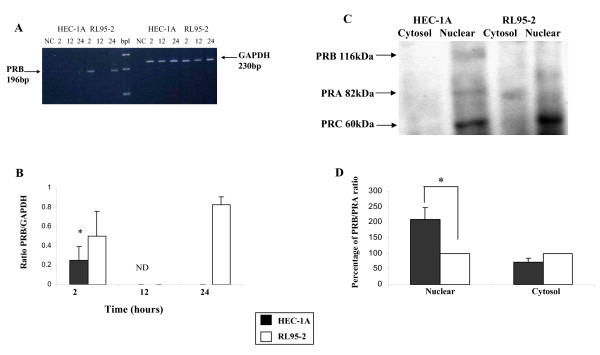
**PR expression in endometrial cell lines**. (A) Transcript expression level of PRB in HEC-1A versus RL95-2 cells. Representative agarose gel of RT-PCR for PRB and GAPDH in HEC-1A and RL95-2 cells. Product size: PRB 196 bp, GAPDH 230 bp. (B) Bar graph describing the mean ± SEM of the ratio between the expression level of PRB/GAPDH, black bars represent HEC-1A, white bars represent RL95-2, * P < 0.05, ND not detected. (C) Representative Western blots from nuclear and cytosol extracts of PRB (116 kD), PRA (82 kD) and PRC (60 kD) expression patterns. (D) Bar graph describing mean ± SEM of the percentage of PRB/PRA ratio in HEC-1A from RL95-2, black bars represent HEC-1A, white bars represent RL95-2, * P < 0.05.

Figure. 1A shows representative 196 bp product of human PRB cDNA. The ratio between the expression level of PRB and GAPDH of each independent experiment from the same cell line under the same treatment was analyzed. The accumulated ratio found to be significantly lower in HEC-1A as compared with RL95-2 cells (ratio 0.084 ± 0.08 versus 0.44 ± 0.13 ratio, respectively, * P < 0.05, Figure. 1B).

In order to further validate our results, we examined nuclear and cytosolic lysates from monolayer of each cell line cultured in the same conditions that were used for spheroids attachment assays. Western blot analysis was conducted using sc-539 antibody against PR. We determined the presence of PR isoforms: the 116 kDa PRB isoform, the 82 kDa PRA isoform and the N-terminally truncated 60 kDa PRC expressed in the cytosol and nuclear fractions. PRB/PRA ratio was calculated for each lane separately. The PRB/PRA ratio in RL95-2 was considered 100 percent. The results are expressed as percent of RL95-2. PRB/PRA ratio in the nucleus of HEC-1A cells was found to be significantly higher as compared with RL95-2 (208% ± 8.8 versus 100% respectively, * P < 0.001, Figure. 1D). In the cytosolic fraction there was no significant difference in the PRB/PRA ratio in HEC-1A cells as compared with RL95-2 (71% ± 12 versus 100% respectively, Figure. 1D).

### The effect of progesterone on spheroid attachment in endometrial cell lines

**Figure 2:**
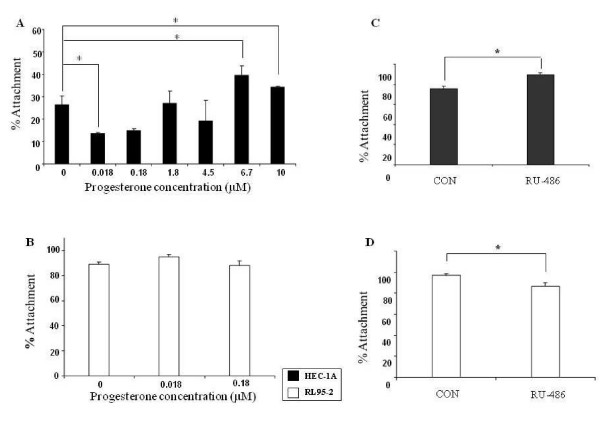
**JAR spheroid attachment to HEC-1A and RL95-2 cells with or without Progesterone**. (A) Bar graph describes mean ± SEM of % attachment to HEC-1A cells treated with progesterone (0–10 μM), * P < 0.05. (B) Bar graph describes mean ± SEM of % attachment to RL95-2 cells treated with progesterone (0–0.18 μM). JAR spheroid attachment to HEC-1A and RL95-2 cells with or without PR antagonist RU-486. (C) Bar graph describes mean ± SEM of % attachment to HEC-1A cells treated with RU-486 (10^-6 ^M). (D) Bar graph describes mean ± SEM of % attachment to RL95-2 cells treated with RU-486 (10^-6 ^M). * P < 0.05.

In order to study the effect of PR stimulation on JAR spheroids attachment to endometrial cell lines, we added progesterone to HEC-1A, the low receptivity cells. A confluent monolayer of HEC-1A cell line was incubated with or without progesterone (0–10 μM) at 37°C and attachment assays were conducted. A total of 1,274 JAR spheroids were divided and examined in HEC-1A cultures treated with different progesterone concentration (n = 239 on treated HEC-1A with 0.018 μM progesterone, n = 258 on treated HEC-1A with 0.18 μM progesterone, n = 233 on treated HEC-1A with 1.78 μM progesterone, n = 185 on treated HEC-1A with 4.5 μM progesterone, n = 94 on treated HEC-1A with 6.7 μM progesterone, n = 67 on treated HEC-1A with 10 μM progesterone, and n = 198 on un-treated HEC-1A cells). As shown in Figure. 2A, we found a dual effect of progesterone, at low concentration of 0.018 μM progesterone significantly inhibited spheroid attachment to treated HEC-1A cells as compared to non-treated HEC-1A cells (13.7% ± 0.47 versus 26.5% ± 3.78, respectively) while a higher progesterone concentration of 6.7 μM and 10 μM increased the attachment rate as compared with non-treated HEC-1A cells (39.8% ± 3.87 and 34.3% ± 0.43 versus 26.5% ± 3.78, respectively, * P < 0.05). RL95-2 is high receptivity cells therefore we tested the low concentration of progesterone, the inhibitory concentrations, (0–0.18 μM). As shown in Figure. 2B JAR spheroid attachment rate to RL95-2 was not affected by progesterone.

### The effect of PR antagonist RU-486 on spheroid attachment in endometrial cell lines

It is well known that progesterone effect is mediated by its cognate receptor – PR. In order to further investigate the role of the PR during the implantation process we tested the effect of PR inhibition on spheroids attachment. We conducted attachment assays to endometrial cell lines treated with RU-486 (10^-6 ^M) PR antagonist. A confluent monolayer of HEC-1A and RL95-2 cell lines was incubated with or without RU-486 for 24 hours before spheroids deliver. In three experiments a total of 270 JAR spheroids were divided and examined in HEC-1A cell cultures (n = 134 on treated cells and n = 136 on non-treated cells) The JAR spheroid attachment rate to HEC-1A treated with RU-486 increased as compared with non-treated cells (89.5% ± 1.7 versus 75.5% ± 2.8 respectively, * P < 0.01, Figure. 2C). In four experiments a total of 302 JAR spheroids were divided and examined in RL95-2 cell cultures (n = 148 on treated cells and n = 154 on un-treated cells). In contrast, in RL95-2 cells, RU-486 decreased the attachment rates of JAR spheroids to treated cells as compared with non-treated cells (96.7% ± 2.1 versus 86.2% ± 3.7 respectively, * P < 0.05, Figure. 2D).

### c-Met expression in RL95-2 and HEC-1A cells

c-Met gene expression was studied by RT-PCR. For normalization we have used the levels of the housekeeping gene GAPDH. In order to exclude the possibility of fluctuation in gene expression during 24 h period, we have studied the basal c-Met gene expression on 2, 12 and 24 h. 2 h after medium replacement considered as starting period (time 0).

**Figure 3:**
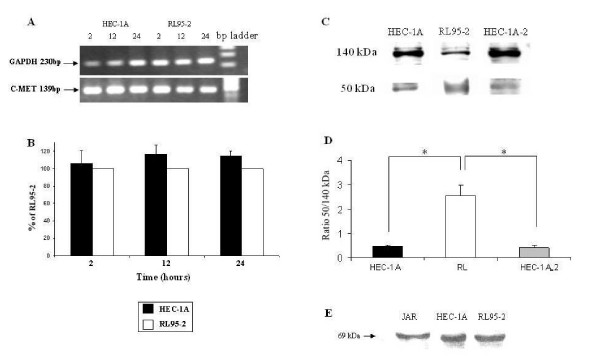
**c-MET expression in endometrial cell lines**. (A) Representative agarose gel of RT-PCR for c-MET and GAPDH in HEC-1A and RL95-2 cells. Product size: c-MET 139 bp, GAPDH 230 bp. (B) Bar graph describing mean ± SEM of the percentage of c-MET/GAPDH ratio in HEC-1A from RL95-2, black bars represent HEC-1A, white bars represent RL95-2. (C) Representative Western blots from whole cell extract of HEC-1A, RL95-2 and HEC-1A-2 cells. Full length of c-MET – 140 kD, short form – 50 kD. (B) Bar graph describing mean ± SEM of the 50 kDa/140 kDa ratio in HEC-1A, RL95-2 and HEC-1A-2, black bar represents HEC-1A; white bar represents RL95-2; gray bar represents HEC-1A-2, * P < 0.05.

Figure. 3A shows representative 139 bp product of human c-Met cDNA. The ratio between the expression level of c-Met and GAPDH of each independent experiment from the same cell line under the same treatment was analyzed. The ratio in RL95-2 was considered as 100%, the results expressed as % of RL95-2 (Figure. 3B). There was no difference in transcript expression level of c-Met between the two cell lines.

In order to study whether c-Met protein is expressed equally in both cell lines, we examined cell lysates from monolayer of each cell line cultured in the same conditions that were used for spheroids attachment assays. Western blot analysis was conducted using sc-161 antibody against c-Met. We determined the presence of two bands in endometrial cell lines (140 and 50 kDa, Figure. 3C). The ratio between the expression level of the short form of c-Met (50 kDa) to the long form (140 kDa) was analyzed for each independent experiment, under the same treatment. In both HEC-1A and HEC-1A-2 the expression level of the 50 kDa form was lower as compared to the 140 kDa form of c-Met. These results are contrary to c-Met's expression pattern in RL95-2 (Figure. 3C). The c-Met forms 50 kDa/140 kDa ratio for HEC-1A and HEC-1A-2 was 0.47 ± 0.07 and 0.43 ± 0.09 respectively, versus 2.56 ± 0.43 for RL95-2, * P < 0.05 (Figure. 3D).

**Figure 4:**
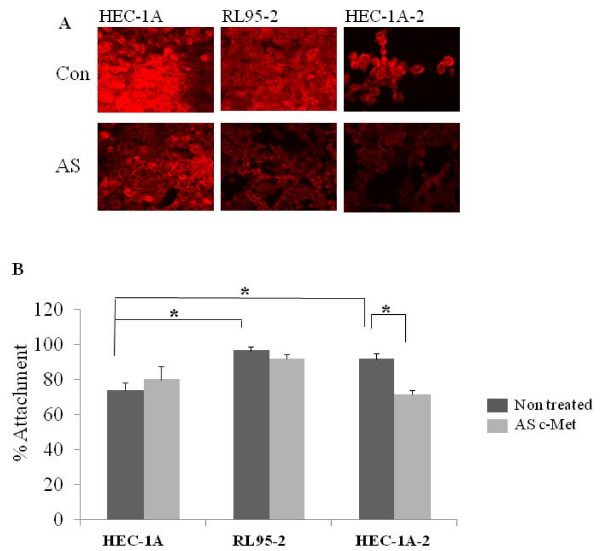
**JAR spheroid attachment to endometrial cell lines, with or without c-MET antisense**. (A) Representative immunoflouroscent labeling for c-MET expression in HEC-1A, RL95-2 and HEC-1A-2 cells with or without c-MET antisense. (B) Bar graph describes mean ± SEM of % attachment (black bars) to HEC-1A, RL95-2 and HEC-1A-2 cells treated with c-MET antisense (grey bars), * P < 0.05.

The expression pattern of HGF, the specific ligand of c-Met, in JAR cells and endometrial cell lines was studied by western blot analysis. As shown in Figure. 4E all the tested cells expressed high level of HGF. This result directed us to perform inhibition of c-Met in order to study the role of the receptor in endometrial receptivity.

### The effect of c-Met inhibition on spheroid attachment to endometrial cell lines

c-Met does not have any known specific inhibitor, so we used c-Met antisense to get the required inhibition. To determine if manipulation on c-Met expression using the antisense (AS) method might alter JAR spheroid attachment to endometrial cell lines we conducted attachment assays to HEC-1A, RL95-2 and HEC-1A-2 cells, transfected with c-Met antisense. Figure. 4A shows immunoflurocense staining for c-Met expression in endometrial cell lines. The c-Met expression level in the transfected cells is lower compared with the un-transfected cells.

JAR spheroids were added to a confluent monolayer of transfected or un-transfected cells and the attachment rates were measured. In five experiments a total of 482 JAR spheroids were divided and examined in endometrial cell cultures (n = 83 on transfected HEC-1A cells and n = 78 on un-transfected HEC-1A cells, n = 82 on transfected RL95-2 cells and n = 82 on un-transfected RL95-2 cells, n = 79 on transfected HEC-1A-2 cells and n = 78 on un-transfected HEC-1A-2 cells). Inhibition of c-Met expression by using the antisense method significantly decreased the attachment rate of JAR spheroids to treated HEC-1A-2 cells as compared to non-treated HEC-1A-2 cells (71.3% ± 2.78 versus 91.7% ± 0.28 respectively, p < 0.01, Figure. 4B). c-Met antisense treatment did not alter the JAR spheroid attachment rate to treated HEC-1A and to treated RL95-2 cell lines (for HEC-1A: 78.2% ± 7.4 versus 72.3% ± 4.5, for RL95-2: 92.2% ± 2.6 versus 96.5% ± 2.6, treated and un-treated respectively, Figure. 4B). As it was expected, according to our previous study (Harduf et al, 2007), the attachment rate of JAR spheroids to RL95-2 is significantly higher as compared to HEC-1A (Figure. 4B).

### Association of PB1 and c-Met

With regard to JAR spheroids attachment, HEC-1A-2, which expressed high level of PB1, was the only cell line that affected following c-Met inhibition. In order to further investigate the possible function of c-Met in the implantation process, we examined if c-Met associates with the intracellular portion of PB1. In six independent experiments, lysates of HEC-1A, RL95-2 and HEC-1A-2 cells were immunoprecipitated for the PB1 receptor (IP: PB1) and immunoblotted for the presence of c-Met (WB: c-Met), using antibodies against c-Met.

**Figure 5:**
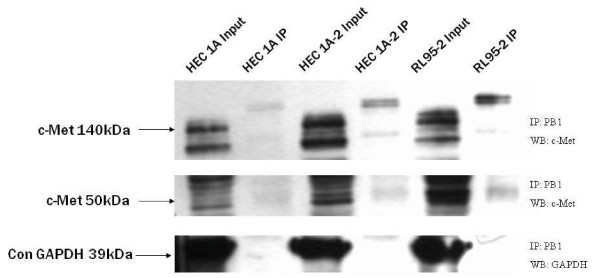
**Immunoprecipitation of c-MET with PB1**. Representative Western blots from whole cell extract of HEC-1A, RL95-2 and HEC-1A-2 cells. Endometrial cells were lysed, and protein in equal amounts (500 μg) was subjected to immunoprecipitation with PB1 antibodies (IP: PB1), followed by Western blotting with c-MET antibody (WB: c-MET). c-MET was observed mainly in immunoprecipitates from RL95-2 and HEC-1A-2. Lower lane represents negative control of PB1 immunoprecipitation, and Western blotting with GAPDH antibody (WB: GAPDH).

As a negative control, PB1 immunoprecipitation was performed followed by Western blotting with GAPDH antibody (WB: GAPDH). As shown in Figure. 5, the higher panel represents the presence of the 140 kDa isoform of c-Met, while the mid panel represents the presence of the 50 kDa isoform of c-Met. The input extract contains all the proteins of the tested cells while the IP extract contains just the proteins associated with PB1. Complexes of PB1 with the 140 kDa isoform of c-Met were observed in HEC-1A, RL95-2 and HEC-1A-2 cells. Complexes of PB1 with the 50 kDa isoform of c-Met were observed in RL95-2 and HEC-1A-2 cells. Complexes of PB1 with GAPDH were not observed in the tested cells (Figure. 5).

## Discussion

Progesterone, estrogen and their cognate receptors are critical and essential regulators of uterine receptivity [34,35]. PR was identified in the nuclei of epithelial cells, stromal cells, and myometrial smooth muscle cells of the uterus. The PR content of endometrial epithelium and stroma varies with the menstrual cycle. The epithelium demonstrated very strong PR expression during the proliferative phase and in the early secretory phase while at the post-ovulation phase it decreased sharply [8]. In general, there was homogeneous expression of the two isoforms in nuclei within the same tissue compartment during most stages of the menstrual cycle, although there were exceptions [36]. However according to another study expression of only one isoform, either PRA or PRB, is common [37]. The mRNA expression of PRB was high in RL95-2 and low in HEC-1A. However, at the protein level the PRB expression was high in HEC-1A and low in RL95-2. Discrepancy between the abundance of RNA molecules and the cognate protein has been recently reviewed [38]. Protein and RNA represent different steps of a multi-stepped cellular genetic information process, in which under differential-regulatory steps they are dynamically produced and degraded. The current study results suggest posttranscriptional modulation of PRB expression in these cells. During the proliferative phase of the menstrual cycle, high levels of both PRA and PRB were noted in the epithelium. The intensity of PR staining increased during the proliferative phase, reaching maximal levels in the mid- to late proliferative phase, and a similar increase in levels of PRA and PRB was evident. By the mid-secretory phase, although overall PR protein concentrations were still further reduced, predominant expression of the PRB isoform was demonstrated [36].

In our study, we found significantly lower PRB protein expression level in RL95-2 as compared with HEC-1A cells. Moreover, the PRB/PRA ratio in the nucleus of RL95-2 cells is significantly lower as compared with HEC-1A. RL95-2 represents receptive epithelial endometrium cells during window of implantation or early secretory phase, while HEC-1A represents epithelial endometrium cells out of the window of implantation. Our results are seemingly contradictory with previous studies, which had demonstrated earlier down regulation of nuclear PRA during early secretory phase in epithelium endometrial cells, while PRB was the predominant isofrm at this stage [36,8]. This difference may be due to the difference in measurement techniques between the two studies.

The distribution of PR subtypes may have important clinical consequences. Both the A and B isoforms of PR are capable of binding progesterone and dimerizing and interacting with progesterone-responsive elements, as well as the transcriptional machinery to regulate gene expression. A growing body of evidence has accumulated in recent years demonstrating that the PRA and PRB proteins are functionally different. PRB has a higher activity in response to progesterone stimulation [39]. In addition, they may regulate different physiological target genes in response to progesterone, and each protein may display different trans-activation capabilities in different target tissues [40]. It is the balance between these two forms that may make it possible for progesterone to affect such diverse physiological targets [41,39].

To facilitate uterine remodeling for embryo attachment, progesterone is known to attenuate estrogen-induced gene expression in uterine epithelial cells. Intriguingly, this suppression is mediated by stromal PR, suggesting that the coordinated action of estrogen and progesterone depends on cross talk between the epithelial and stromal compartments of the uterus. The mechanism by which progesterone suppresses estrogen's action remains poorly defined [35]. PRA was implicated as the more important mediator of stromal- epithelial interaction [41]. More recently, selective ablation of the PRA and PRB proteins in mice had facilitated examination of the contribution of the individual PR isoforms to the reproductive activities of progesterone. Ablation of PRA results in severe abnormalities in ovarian and uterine function leading to female infertility, whereas ablation of PRB does not affect either ovarian or uterine function. The anti-proliferative and anti-inflammatory roles of PR, both of which are anti-estrogenic, may be imparted primarily by the A protein [39]. Thus, PRA is both necessary and sufficient to elicit the progesterone-dependent reproductive responses necessary for female fertility [42]. This report is consistent with our finding that PRA is the dominant isoform in RL95-2.

In order to study the involvement of PR in spheroid attachment to endometrial cell lines, we stimulated PR with progesterone. A dual progesterone effect was found in HEC-1A cells (low concentration of progesterone significantly inhibited spheroids attachment, while higher concentrations of progesterone increased attachment rates to HEC-1A cells). It is well known that progesterone function in regard to trophoblast-endometrium interaction is dose-dependent [43] and that the progesterone response is biphasic [44].

Attachment rates of JAR spheroids to RL95-2 were not altered in response to low concentrations of progesterone. It might be explainable by the different dominant PR isoform in those cells: PRA in RL95-2 and PRB in HEC-1A cells.

Progesterone is known to control cell adhesion proteins including ECM proteins and their cellular receptors, integrins. It was shown that progesterone upregulates the expression of these molecules [45,46]. It might be suggested that progesterone's action in increasing the attachment rate occurs via integrin stimulation.

In our study, PR antagonist RU-486 appeared to decrease the JAR spheroid attachment rate to RL95-2 cells by 10%, while it increased the attachment rate of JAR spheroids to HEC-1A cells by 18.5%. In accordance with our study it was shown that RU-486 inhibits the expression of endometrial receptivity markers (LIF, Integrin α_V_β_3_, MUC1) and also inhibits attachment of human embryos to an in-vitro endometrial construct [47,48]. It was shown that the potent progesterone antagonist RU-486 has the potential to acquire substantial agonist activity in response to stimulation of cAMP signaling pathways. Moreover, agonist activity appears to be the result of authentic RU-486 activity through the PR [49]. There is significant implication for the PR subtype in regarding to RU-486 effect. It was documented that the ratio of PR isoforms B/A strongly influences the direction of a tissue's response to progesterone antagonists [50]. It might be suggested that RU-486 acts differently on each of the endometrial cell lines. In HEC-1A the dominant isoform is PRB. Inhibition of it by RU-486 changed the ratio PRB/PRA and elevated the relative amount of PRA, which seemed to be involved in the attachment process. Nevertheless it requires further investigation.

The expression pattern of c-Met and its biological role have been studied in various tumors, but, as yet, these findings have not been incorporated into the implantation studies. We undertook this study to characterize the expression pattern of c-Met and to determine whether c-Met is a potential target for trophoblast epithelial interaction. There was no difference in transcript expression level of c-MET between the two cell lines. However at the protein level we found a different expression profile in the two cell lines. The ratio of 50 kDa/140 kDa of c-Met isoforms was found to be significantly lower in the HEC-1A cell line as compared to RL95-2. The c-Met expression pattern in HEC-1A-2 was similar to the pattern observed in HEC-1A. Nevertheless, the ratio 50 kDa/140 kDa of c-Met isoforms were lower in the first.

The Met proto-oncogene encodes a transmembrane tyrosine kinase of 190 kDa (p190MET), which has recently been identified as the receptor for hepatocyte growth factor/scatter factor (HGF). p190Met is a heterodimer composed of two disulfide-linked chains of 50 kDa (p50 alpha) and 145 kDa (p145 beta). It is believed that the truncated 50 kDa form is a cytosolic form called cyto-Met [51]. This form is suggested to be degraded by rapidly polyubiquitinated [52]. It was also hypothesized that the production of the C-terminal truncated Met forms may have a physiological role in modulating the Met receptor function [53]. They interfere with the Met receptor signal transduction pathway by competing with the intact receptor for binding to the ligand. Such a negative regulatory role has already been shown. Moreover, the transmembrane truncated receptors, devoid of tyrosine kinase activity, may form inactive heterodimers with the intact receptors [54,26].

It was suggested that in mouse the presence of the shorter variant transcript and its corresponding protein isoform in a variety of normal tissues has a unique physiological role [55]. Thus it might explain our observation of a different expression profile of the two forms of c-MET receptor between the cell lines, which differ in their receptivity potential.

According to our results, c-Met AS was sufficient to inhibit spheroid attachment to HEC-1A-2 cells but not to HEC-1A or RL95-2. The only difference between RL95-2 and HEC-1A-2 is the higher expression level of the truncated c-Met form and between the HEC-1A and HEC-2A is the higher PB1 expression level in the latter. It was published that cells expressing the endogenous proteins, PB1 and c-Met, associate in a complex [56,57,15]. In addition, binding of Sema 4D to PB1 stimulates the tyrosine kinase activity of c-Met, resulting in tyrosine phosphorylation of both receptors [15]. This suggests that integration of cell-restricted expression of receptor partners that modulate kinase outputs with the intrinsic signaling features of receptors is required for specification of biological responses. In this study we suggest that c-Met interacts with PB1 in all endometrial cell lines. However, a high interaction was observed in HEC-1A-2 and RL95-2, which is not surprising since PB1 expression level is significantly higher in these cell lines. However this scenario is probably more complex because plexins can interact with a variety of growth factors and receptors [58].

## Conclusion

Attachment and invasion processes seem to be separately regulated. The findings of differential c-Met and PR profile expressed during the receptivity period may have important implications. PRA is most probably involved in endometrial receptivity and in the attachment process. c-Met is creating a complex with PB1 contributing to the regulation of endometrial receptivity and the attachment process.

**Figure 6:**
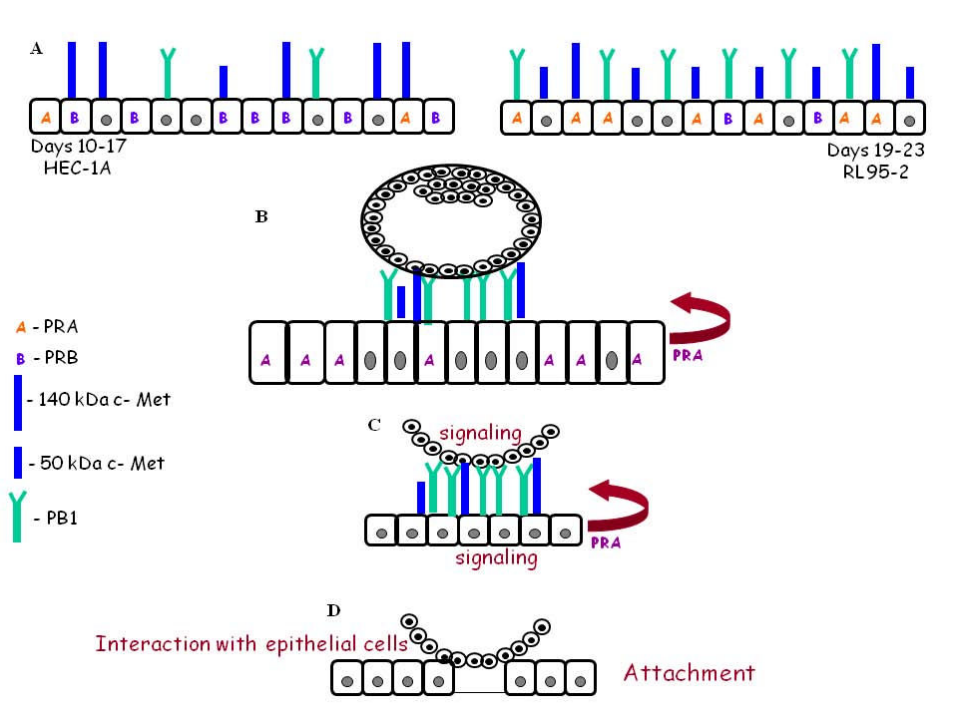
**Proposed hypothesis of epithelial endometrial receptors role in endometrial receptivity and attachment stage**. (A) Different expression pattern of endometrial receptors (PR, c-MET, PB1) in endometrial cells at the window of implantation and in endometrial cells before the window of implantation. (B) Attachment stage is mediated by epithelial endometrial receptors: PRA, hetrodimer of PB1 – c-MET (mainly 140 kDa form) and homodimer of PB1 (C) Signal transduction is activated in the embryo and endometrial cells. (D) Interaction of trophoblast embryo cells with epithelial endometrial cells leading to attachment and to implantation.

We hypothesized that before cells attachment, an interaction between the endometrial blastocyst receptors occurs. Consequently cell-cell attachment followed by cell adhesion open the door for the beginning of successful implantation (Figure. 6)

## Competing interests

The authors declare that they have no competing interests.

## Authors' contributions

HH carried out the laboratory work, participated in design of the study, performed the statistical analysis and drafted the manuscript. SG Participated in conceiving and designing of the study, directed the laboratory work, helped in the statistical analysis and in drafting of the manuscript. ES Conceived and design the study and edited the manuscript. All authors read and approved the final manuscript.
